# Ontological modeling of the International Classification of Functioning, Disabilities and Health (ICF): Activities&Participation and Environmental Factors components

**DOI:** 10.1186/s12911-021-01729-x

**Published:** 2021-12-29

**Authors:** Silvia Cozzi, Andrea Martinuzzi, Vincenzo Della Mea

**Affiliations:** 1 Insiel S.P.A., Udine, Italy; 2 Conegliano-Pieve di Soligo Research Centre, IRCCS “E. Medea” Scientific Institute, Pieve Di Soligo, Italy; 3 Department of Mathematics, Computer Science and Physics, University of Udine, Via delle Scienze 206, 33100 Udine, Italy

**Keywords:** ICF, Ontology, Ontology engineering, Biomedical classifications

## Abstract

**Background:**

The International Classification of Functioning, Disability and Health (ICF) is a classification of health and health-related states developed by the World Health Organization (WHO) to provide a standard and unified language to be used as a reference model for the description of health and health-related states. The concept of functioning on which ICF is based is that of a “dynamic interaction between a person’s health condition, environmental factors and personal factors”. This overall model has been translated into a classification covering all the main components of functioning. However, the practical use of ICF has highlighted some formal problems, mainly concerning conceptual clarity and ontological coherence.

**Methods:**

In the present work, we propose an initial ontological formalization of ICF beyond its current status, focusing specifically on the interaction between activities and participation and environmental factors. The formalization has been based on ontology engineering methods to drive goal and scope definition, knowledge acquisition, selection of an upper ontology for mapping, conceptual model definition and evaluation, and finally representation using the Ontology Web Language (OWL).

**Results:**

A conceptual model has been defined in a graphical language that included 202 entities, when possible mapped to the SUMO upper ontology. The conceptual model has been validated against 60 case studies from the literature, plus 6 ad-hoc case studies. The model has been then represented using OWL.

**Conclusions:**

This formalization might provide the basis for a revision of the ICF classification in line with current efforts made by WHO on the International Classification of Diseases and on the International Classification of Health Interventions.

## Introduction

The International Classification of Functioning, Disability and Health (ICF) is a classification of health and health-related states developed by the World Health Organization (WHO) since 2001 [1]. The overall aim of the ICF classification is to provide a standard and unified language to be used as a reference model for the description of health and health-related states. The ICF complements WHO’s International Classification of Diseases (ICD), which contains information on diagnosis and health condition, but not on functioning status, and with it ICF constitute the core classifications in the WHO Family of International Classifications (WHO-FIC).

The concept of functioning on which ICF is based is that of a “dynamic interaction between a person’s health condition, environmental factors and personal factors” [2]. The description of the functioning states takes into account factors affecting them, giving a biopsychosocial model of disability, seen as a combination of two sets of factors, physical (body functions and structures, with their impairments) and contextual (environmental and personal factors, seen with the role of facilitators or barriers), that can limit activity and restrict participation in social life. This overall model has been translated into a classification covering all the main components of functioning.

However, the practical use of ICF has highlighted some formal problems, mainly concerning its contents, as the definition of some of its classification items may be seen conceptually unclear or ontologically incoherent, or some interest areas seem not adequately covered.

According to classification theory, one of the classification criteria aimed at conceptual clarity is mutual exclusivity: a given thing can belong to only one category. As an example of violation of the mutual exclusivity criterion in the Activities and Participation component, some intentional behaviors are currently classified within the Body Functions component (“b3401 Making a range of sounds”), while others within the Activities and Participation component (“d330 Speaking”) [3]. A further example is the writing activity: it is described as an element in itself (“d170 Writing” in the block “Applying knowledge (d160-d179)”) and accompanied by a purpose (“d345 Writing messages” in the block “Communicating—producing (d330-d349)”). In this case the problem is mainly terminological since the description "Writing messages" taken alone does not clearly refer to the communication context.

As for ontological coherence problems, Activities and Participation chapter 2 “General tasks and demands” resembles a classification of tasks based on complexity and other features while in the other parts of the component are described concrete activities [4]. Incongruent classification is present in the chapter 2 mentioned above, where two categories are distinguished on the basis of multiplicity (“d210 Undertaking a single task” and “d220 Undertaking multiple tasks”) while two on psychological demands (“d230 Carrying out daily routine” and “d240 Handling stress and other psychological demands”). Within the body function domain some manifestations of functioning impairments are listed as inclusions (or as more recently they have been renamed “remarks”), like myopia or color blindness for “b210 Seeing functions”, and others as autonomous categories, such as “b2401 Dizziness”.

Regarding domain coverage, environmental factors classification seems not exhaustive, and a lack of many relevant items, such as factors related to the working environment, has been suggested [5].

Aim of the present work is to provide an initial ontological formalization of ICF beyond its current status, focusing specifically on the interaction between activities and participation and environmental factors. According to the overall model adopted for ICD-11, also for ICF there could be two levels: a Foundation, including all entities organized according to semantic principles, and a Linearization level, corresponding to the proper classification to be used in practice, which is derived from the Foundation. The present work is aimed at modeling part of the Foundation level to provide a more robust linearization level.

The formalization conducted in this study concerns both the model and the structure of the ICF. The definition of an ontology for ICF will help to identify unclear or inconsistent contents so that concrete improvements on the classification will be easily applied. The ontological representation of the ICF is the necessary pre-requisite for its computerized management and a complete computerization of the ICF would bring exceptional help in its daily clinical use. Moreover, the logical rearrangement of the internal relationship among categories within a given domain and among different domains would answer the frequent questions that arise when choosing the most appropriate category to describe an observed functioning.

## The current status of ICF

### ICF model and structure

The ICF provides an interactive conceptual model in an attempt to integrate the individual and social model of disability into the biopsychosocial model. The ICF model shows relationships between six dimensions: health conditions; three functioning dimensions: body factors (body functions and structures), activities (abilities to perform actions) and participation (the experience of being part of society); two contextual factors dimensions: environmental and personal factors.

**Fig. 1:**
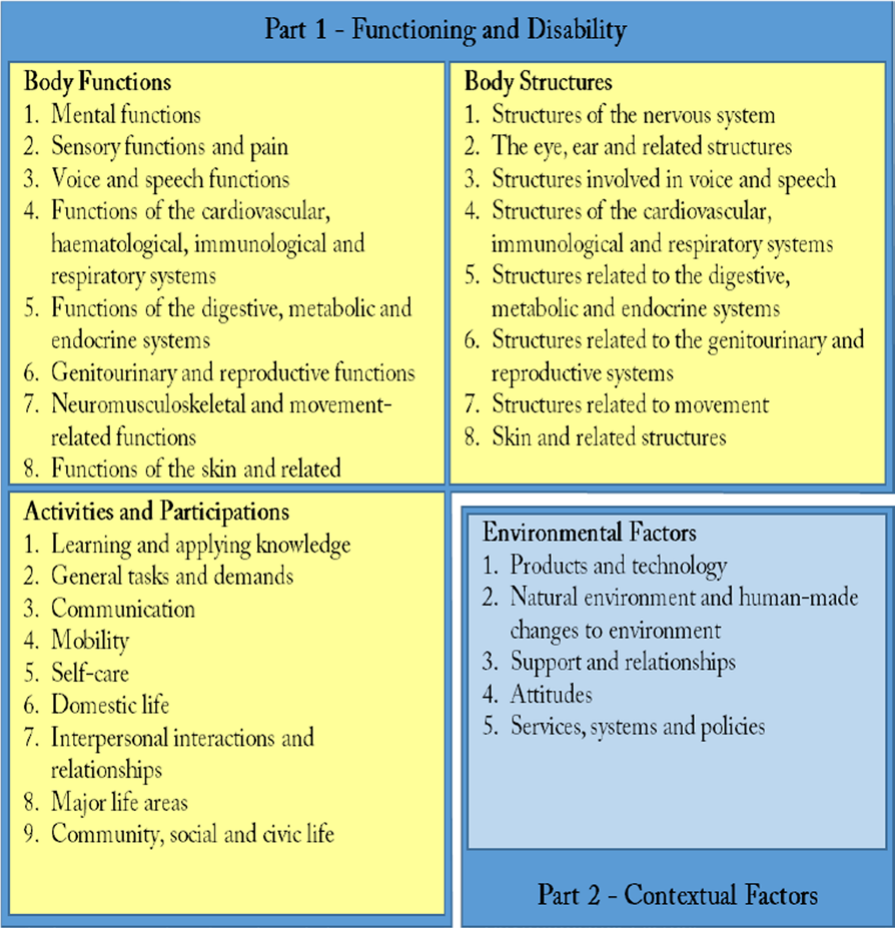
Overall ICF structure: components and domains/chapters

The classification system of the ICF has two parts, Part 1-Functioning and Disability and Part 2-Contextual Factors, each with two components subdivided hierarchically into chapters, blocks and categories, the classification units, that are identified by alphanumeric codes and described by textual definitions. The areas covered by the four components concern health and health-related domains. These are sets of related physiological functions (Body Functions component), anatomical structures (Body Structures component), actions, tasks, areas of life (Activities and Participation component), and external modulators (Contextual Factors component). The ICF has a separate chapter for each of the domains as listed in Fig. 1.

### Measurement: the ICF qualifiers

To describe the level of functioning the ICF provides qualifiers. Qualifiers are sets of predefined values used to measure specific qualities of functioning: each value is identified by a code and a description and is used to record the extent of functioning or disability in a domain or category, or the extent to which an environmental factor is a facilitator or barrier. The current version of ICF lists seven qualifiers: impairment, nature of change, localization, performance, capacity, barrier, facilitator. ICF states which qualifier should be used with each component, since the measured qualities may be pertinent only to some domains.

### Related work

In the last few years an effort is ongoing to harmonize the whole Family of International Classifications maintained by WHO [6], which include the International Classification of Diseases (ICD), the forthcoming International Classification of Health Interventions (ICHI), and ICF itself. Harmonization will include a better specification of the concepts expressed by the 3 classifications, as well as the relationships among them. However, both the latest revision of ICD, ICD-11, and ICHI have been subject of some formalization effort [7, 8], while ICF until now remained almost untouched, although some limits started to be noticed.

In fact, the use of ICF has highlighted some formal problems, mainly concerning its contents. As problems in using ICF were highlighted, works investigating the contents of the classification pointed out the need to improve conceptual clarity and ontological coherence [3, 4, 9], as well as to extend the domain covered by the classification [5]. Other shortcomings related to the Body Functions and Body Structure components are detailed in [10]. From conceptual problems, practical difficulties often arise in the use of the ICF by professionals, with consequences in the quality of the coding of assessments, possible resistance to use the ICF and difficulties in sharing of knowledge.

The creators of ICF are aware of the problems highlighted and have taken some initiatives to address them and to define corrective actions of improvement. Two workshops on ICF ontological aspects were organized, in 2008 in Nottwil (Switzerland) and in 2010 in Venice (Italy), and a restricted work group was created in 2015 to formalize problems and discuss solutions. The shared vision is that improvement would be gained if the ICF was based on a clear semantic reference model and if ontological approaches were adopted to guide its development process. Until now only marginal problems have been addressed but no overall solution has been outlined. Studies have been held mainly on categorial structure [11] and content model [6], with attention for enhancements to the overall scheme of ICF [12].

### Ontology engineering

There are currently many methods for ontology engineering and ontology development [13, 14]. All the methods are composed of different activities with a development process that is not linear but follows a cyclic approach, with recursive incremental cycles of refinement steps, in which each activity can be repeated several times [13]. In many methodologies, the main activities oriented to ontology development can be grouped into pre-development, development and post-development activities. As documented in [15], the main steps of the ontology development are usually scope definition, knowledge acquisition, conceptualization, formalization and evaluation and, according to [16, 17], can be further decomposed as follows:

1. Determine the domain and scope of the ontology
2. Consider reusing existing ontologies
3. Enumerate important terms in the ontology
4. Define the classes and the class hierarchy
5. Define attributes and relationships
6. Define the restrictions of the properties
7. Create instances

## Methods

For this study, the main steps of the ontology development process documented in [16, 17] have been considered. Furthermore, some guidelines from the METHONTOLOGY method [15] were followed:

- Most of the knowledge is acquired at the beginning of the ontology construction.
- The integration of other ontologies is not postponed to the implementation activity but should be carried out at the knowledge level.
- The ontology conceptualization must be evaluated accurately to avoid propagating errors in further stages of the ontology life cycle.

**Fig. 2:**
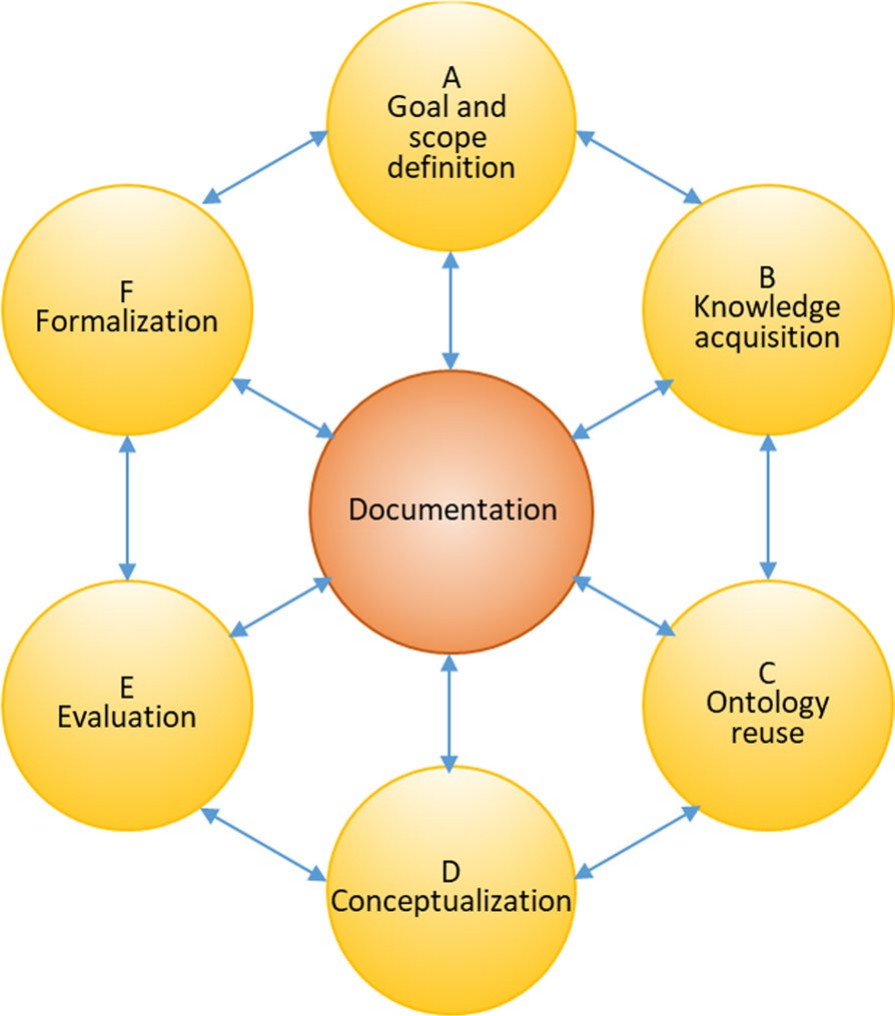
The ontology development method employed in the current study

The activities and phases of ontology development can be identified also in software engineering and in general in software development lifecycle management methodologies. The TOGAF framework [18], a detailed method and a set of supporting tools for developing an enterprise architecture has been considered in the study. Taking into account the characteristics of this study for which a high-level methodology seemed more appropriate [19], the TOGAF model was simplified and a practical approach of ontology development was followed, as pictured in Fig. 2 where the final stage is the ontology formalization, as it is the main goal of the study.

A central part of the methodology adopted is the documentation. All contents not specific to a single phase but to be shared among phases were registered and kept in a shared repository. The repository, simply a directory in the hard disk, was defined along with naming and versioning rules of the artifacts produced.

### Goal and scope definition

In order to scope the study, a template of the Ontology Requirements Specification Document was followed, with the use of competency questions to state high level requirements. The study constraints where clearly specified along with its contents and a simple stakeholder profile to guide the direction of the ICF ontology specification.

### Knowledge acquisition

In this phase, performed in the early stages of the study, many sources of information were selected and the materials collected were classified for further use and reference.

The main knowledge areas identified to be covered were about the ICF (history, principles, model, structure, contents, usage, limits, etc.) and ontologies (principles, types, constituting elements, supporting ICT technologies, etc.). To cover the ICF domain materials officially related to the ICF were used, such as the ICF definition and manual, checklist, case studies, etc., and WHO-FIC annual meeting posters. Where needed, ICF domain experts were involved to clarify shadow areas and to discuss and compare ideas. As for scientific articles were privileged those about the ICF explanation, structure problems, usage experiences, alternative model proposals in order to gain a broader, deeper and practical knowledge about the domain and to support the decisions during the formalization of the ICF. Knowledge about ontologies was acquired mainly from technical materials and from documentation about well-known ontologies.

The understanding about the domain was documented using a “statement log”, a list of statements that describe the domain. In the statement log each statement was classified according to the domain characteristic. The statement log, enriched mainly in this phase but also in subsequent ones, was the source of knowledge about the domain from witch relevant terms were collected.

To perform the collection of terms, the term beating technique was followed: each statement was analyzed and nouns and verbs were extracted as candidate concepts and relations. The goal was to select and collect a broad variety of associated terms, leaving the evaluation of their relevance for later. A "term tracker" was created to support this work: a list of structured information about the main terms identified using the statement log or derived from the study of the domain. Each term was recorded in a term pool, with a description, the source reference, the prospective entity in the ontology (i.e. class, relation or individual), synonyms, arguments for relations, the action to be performed on the term (i.e. promote, discard or pending) and some notes. The aim was to record all the relevant terms and to decide about their usage, and possibly change the decisions, without actually deleting any term. This method is consistent to the iterative approach where gaining more knowledge may require to change previous choices.

### Selection of the upper level ontology for mapping

A requirement of the study was to map the ICF ontology to an upper level ontology: BioTop [20] or SUMO [21]. To evaluate which one to use in the mapping, their content was analyzed in a comparative way with a prototyping technique: a draft model of the ICF ontology was sketched using the terms already identified and was used as a benchmark against which to compare the contents of the two ontologies.

### Conceptual model definition

Given the complexity and criticality of the subject matter, a specific method based on those used in computer science was adopted in this phase. First of all, the contents of the conceptual model to be released in the phase (classes, subsumptions, relations, individuals and multiplicities) and conventions were established to ensure consistency of the model, then the representation language to be used in the model definition and, finally, the process to follow in the conceptualization.

Some modeling languages especially designed for ontologies or that have specific extension for knowledge modeling or ontology design were evaluated, such as Unified Modeling Language (UML) [22], IDEF5 (Integrated Definition for Ontology Description Capture Method) schematic language [23], the graphical language used in the book [24], Visual Notation for OWL Ontologies (VOWL) [25]. For the graphical representation of the ontology we chose to use VOWL version 2, whose primitives and specifications are found in its specification language manual [26].

The process adopted during the analysis followed the principles of domain decomposition and separation of concerns, in accordance with the Domain-Driven Design approach [27]. A top level model was initially defined where the domain to represent was divided in sub domains and dependencies among sub domains were specified. The conceptualization then proceeded analyzing each sub domain separately, identifying classes and relations and their mapping towards SUMO, with a harmonization phase at the end of every iteration to integrate each conceptualized sub model. When all sub domains were conceptualized, a review was held to compose the final model.

### Conceptual model evaluation

In order to avoid propagating errors in subsequent stages of the ontology development, the evaluation phase was performed as soon as a part of the conceptual model was specified, following the approach of Model-based testing [28] that allows to gain benefits of early testing thanks to the execution of functional tests at the same abstract level as the model. The evaluation concerned validation aspects and was made against case studies, some specially prepared in order to validate specific parts of the model, and other officially defined for the ICF. The validation phase was repeated many times, until the model was considered well defined and no more modifications to the model were identified.

### Formalization

The final phase, the formalization of the ICF ontology in OWL, was executed only after the model evaluation. To represent the ICF ontology in the OWL formal language was used Protégé [29], a free, open-source ontology editor.

## Results

The two ICF components Activities&Participation and Environmental Factors were thoroughly analyzed and compared to BioTop and SUMO upper level ontologies; SUMO was chosen as the reference ontology. Two different but correlated models were developed: the domain model, with the main concepts of the ICF and the relations among them, and the content model, to formally represent all ICF classification knowledge in a systematic and structured way.

The models were initially conceived at conceptual level, represented with a non-formal visual language (VOWL2), and were validated before formalization. Both were finally formalized in OWL and an ontology of the studied ICF subdomain was created, where the two models were merged. Thanks to the design choices, the obtained ontology is compatible with the whole ICF even if it represents a subdomain.

In the following sections, details will be given regarding some specific steps of the ontology development.

### Goal and scope definition

Firstly, the intended end users of the ICF ontology were identified: coders (nurses, physicians, therapists, etc.), classification experts and the WHO organization. Brief profiles of the stakeholders have been developed, including role, expectations, impact and risk level.

Secondly, since the development of the ICF ontology is motivated by the scenarios related to its use, we identified 7 main scenarios, as follows.

1. Search the ICF codes to be used in the assessments or in order to manage the ICF coding scheme.
2. Search examples, in order to select the right code to use.
3. Identify the position of a category in the taxonomy and navigate in the categories hierarchy.
4. Describe more accurately the categories of the ICF.
5. Find similar categories.
6. Group ICF assessments data in different ways.
7. Identify and exploit the major relationships between activities and environmental factors, activities and body functions, activities and body structures, activities and activities.

Finally, we identified 4 non-functional and 20 functional requirements. The latter have been provided in form of competency questions, as questions to which stakeholders would like to find an answer using the ontology of the ICF. Competency questions have been grouped according to the end users. Examples of competency questions are as follows:

- Are there any activities that can be differently assessed according to their articulation in phases (start, continue, end)?
- Are all the activities represented in the Activities and Participation categories intended to be executed many times or are some of them assessed as executed only once, as a single activity?
- Is there any correlation between the activities of the ICF Activities and Participation component? For example, if a subject is not in a position to perform an X activity, then he cannot be able to perform even the Y activity: if a person has significant difficulty in moving, he should not be able to assist other people in their movement.
- How capacity can be established, which is by definition a theoretical construct?
- How can the effect of environmental factors on body functions be described (e.g. the effect of a drug)?
- Shall we expect a consistent criterion (from whole to part, from initial to final, from early to late, etc.) in going from categories at higher level (e.g. second level categories such as b210) and higher level categories (e.g. third and fourth level)?

However, please note that some questions can be answered only with a more extensive formalization of the classification, of which the work presented here is only a first step.

### Knowledge acquisition

The knowledge gained about the ICF useful to build the ontology was strictly organized in order to facilitate the formalization phase. The outcome of the knowledge acquisition step included two artifacts: the statement log, with a collection of 72 statements about the domain, and the term tracker, a structured register of selected terms with 83 elements.

### Selection of the upper level ontology for mapping

The results of the initial mapping on a draft ICF model showed that BioTop, as a domain ontology especially oriented to biomedicine, was too specific in respect to the domain to represent, so SUMO was selected. One reason is that ICF follows a biopsychosocial model, which tries to be more general than a medical approach to disability.

After refinement of the draft model, 72 entities from SUMO have been investigated and 35 were candidate to mapping. At the formalization phase, 27 mappings to SUMO entities were left.

### Conceptual model definition

This important phase produced three outcomes: the conceptual model of the ICF ontology in a graphical language with the contents established, the recording of all the SUMO terms used in the mapping and a model repository of all its elements. The latter is a collection of all the entities of the conceptual model where, for each entity, the definition, the type (class, relation or individual), the mapping to SUMO, the arguments of relations, the status (draft, defined, discarded) and some notes are recorded. In the repository we recorded 202 entities.

The conceptualization of the ICF ontology was held on an iterative basis with a top-down approach and required cycles of refinement with in-depth analysis of concepts, acquisition of domain knowledge and sometimes involvement of domain experts.

#### Main concepts elicitation

To specify the initial model of the ICF, the main concepts that define it were identified using the ICF definition documentation [1], where in the first chapter there is a high level definition of the ICF. Using that source of information, its contents were reformulated and a synthetic definition of the ICF was provided:

The ICF classification is a framework for the description of health and health-related states. The latter are evaluated measuring relevant qualities against health and health-related domains and measuring the influence of contextual factors, in general or related to those domains.

The first two concepts that emerge from the previous definitions are “framework” and “health and health-related state”, the former expressing the static structure of the ICF classification and the latter referring to dynamic information about health and health-related states of human being, classified with the ICF structure. The concept identified with the framework term refers to all the “classification units”, ICF entities used in the classification. Thus to represent the ICF framework, we chose the name “ICF Entities”. A third concept regards the measure of qualities and influence of contextual factors. This is done using qualifiers, so a specific bounded context is identified for this part of the domain.

The other relevant concepts that compose the structure of the ICF were derived from its scope and contents [1], summarized in the statement log. Health and health-related domains, more simply called “Health Domains”, are exhaustively partitioned into three components which can be considered different sub domains, or contexts, and therefore studied separately: Body Functions, Body Structures and Activities and Participation. The ICF has a fourth component, named Contextual Factors, which is divided into Environmental Factors and Personal Factors sub contexts. Personal factors were not classified in the ICF at the time of this study, but its concept is explicitly identified in ICF denoting its relevance. Therefore the concept of personal factor was included in the model as an evolving point, without further formalization.

The ICF defines qualifiers that are used to measure some characteristics of human functioning or disability. Qualifiers are applied to categories (health and health-related domains categories and environmental factors categories) in order to specify their qualities. The association of qualifiers values to categories is the aim of an ICF observation.

The concept “health and health-related state”, that in the model was represented by the short form “Health States” to improve readability, can be further detailed considering the coding conventions provided in [1]. A health and health-related state is a snapshot of the functioning of a human being, obtained by using the qualifiers to measure:

- his/her functioning in respect to the categories of the three first components of the ICF;
- the influence of contextual factors on his/her functioning.

Even if the main ICF documentation [1] uses the term health state, that concept refers to the broad meaning of functioning status or situation, as health states in ICF can be described as the capacity to carry out a set of tasks and actions besides changes in body functions and/or structures arising from a health condition [30]. Despite the term functioning situation seems more appropriate and envisioned in the future revisions of ICF harmonized with the other WHO-FIC [6], in this study we adhere to the current ICF terminology and use the term health state.

#### Relationships among bounded contexts

The Domain-Driven Design principles were used to separate the concerns of the large and complex domain of the study into bounded contexts. In the meantime, relationships among the bounded contexts were highlighted as contact points between them, that is dependencies that exists and must be carefully handled. Unlike Domain-Driven Design, in this study patterns were not applied, but the dependencies were discovered and annotated for future usage.

**Fig. 3:**
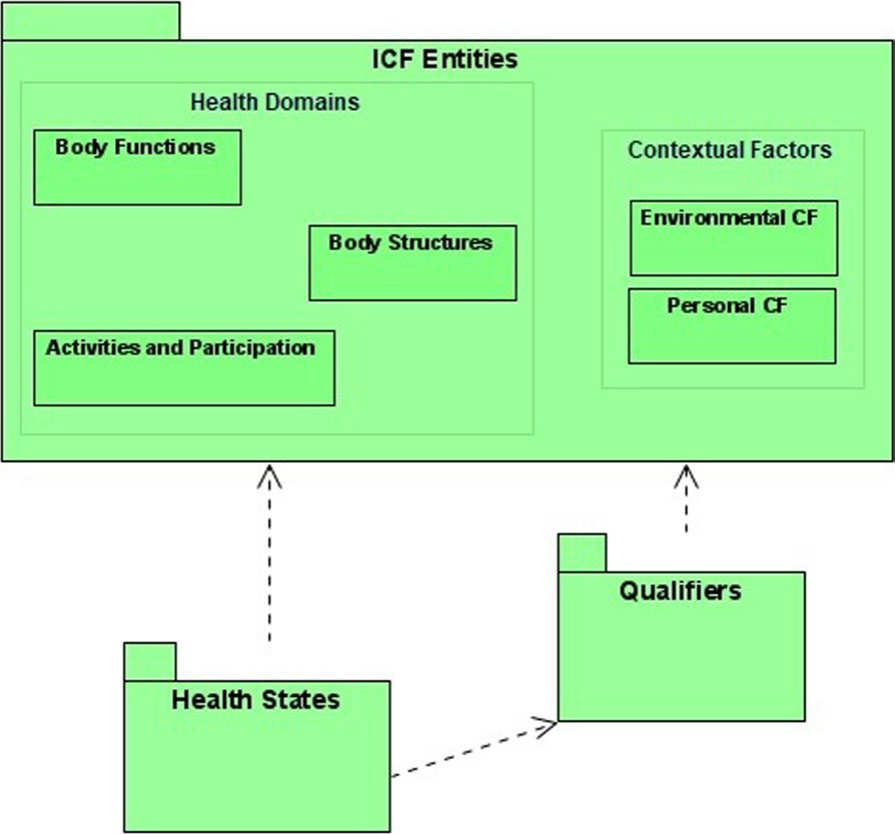
The context map of the ICF as the initial conceptual model with the relationships among contexts

The ICF Entities context is related to the Qualifier context, since qualifiers are applied differently to ICF classification units. The Health States context is related to the ICF Entities context and the Qualifiers context, since health states are described using ICF entities and Qualifiers. In the relation between ICF Entities and Qualifiers, the first may influence the latter because changes in the entities may require updates to qualifiers. Similarly, health states descriptions need the other two contexts which, consequently, may influence it. The initial conceptual model of the ICF with the relationships among contexts is presented in Fig. 3, where the UML component diagram notation is used. The direction of the arrows shows the verse of influence: the point is the influencer.

This initial model was used as a guide and a map for the analysis of the entire domain which was detailed following an iterative process that brought out concepts and their relations. Firstly, the analysis focused on elements inside each bounded context in order to make clear their structure and semantics. Then attention has been paid on contact points between bounded contexts and were addressed inter context relations. Finally, a general assessment has been performed to compose a consistent model. The ICF components Body Functions and Body Structures have been left out, so their qualifiers, as they were not part of the scope of this study.

#### ICF entities

The bounded context ICF Entities represents the structure of the ICF classification: it was modeled with the class *ICFCategory* that includes all the categories of the ICF framework. It was mapped to SUMO Taxonomy class, which is a type of *ClassificationScheme*.

The entire ICF category hierarchy for its four components has been implemented by the *ICFCategory* class; the individuals of this class are categories, blocks and chapters that make up the classification scheme of the ICF. Body Functions (*BodyFunction* class), Body Structures (*BodyStructure* class) and Activities and Participation (*ActivityAndParticipation* class) components were grouped under the class *HealthDomain*. Contextual Factors component (*ContextualFactor* class) was distinct from health domains because it represents a different concept: it is the set of elements that may have an influence on health domains while health domains are sets of elements related to health and well-being.

#### Qualifiers

The analysis of the Qualifiers context initially covered all the qualifiers of the ICF, not only those of the scope of this study (Activities and Participation and Contextual Factors components), and narrowed the perimeter to its scope later, when the model of the context had been sketched.

The concept behind qualifiers refers to qualities, or characteristics or aspects that the ICF wants to measure. ICF declares the values that should be collected with each qualifier. In order to make assessments as objective as possible, the ICF extends the values with a short description and with a percentage range, creating a set of intervals that cover all the possible extent.

Qualifiers values are predefined sets of such values. In ontology languages, such as OWL, sets of predefined values can be represented in two ways: datatypes or classes with individuals. In OWL, datatypes are entities that refer to sets of data values [31]. Thus, datatypes are analogous to classes, the main difference being that the former contain data values such as strings and numbers, rather than individuals. The other possibility to represent value sets is to use classes where values are provided in the form of their individuals.

The first option, using datatypes, seems the easier one but the second presents greater flexibility and openness to future scenarios. In fact, in order to say anything about qualifiers values, they need to be individuals. For instance, when qualifiers values are individuals it is possible to give them additional types (i.e. it is possible to make groupings of values) and then to ask a count of the new types. It is not possible to do this if qualifiers values are literals, because literals can't be the subject of statements [32]. In the conceptual model, qualifiers values have been represented with individuals in enumerated classes [33]. These are classes defined by precisely listing the individuals that are the members of the class using the *owl:oneOf* property.

We identified some SUMO classes to use in the definition of the conceptual model of qualifiers.

- *Attribute*: a quality, an abstract entity that is exhaustively partitioned into internal and relational attribute, such as a qualifier.
- *SubjectiveAssessmentAttribute*: an attribute which lacks an objective criterion for its attribution, i.e. the attribution of this attribute varies from subject to subject and even with respect to the same subject over time.
- *Difficulty*: as the quality of being difficult, is considered a subclass of SUMO *SubjectiveAssessmentAttribute*.

The sub model for qualifiers created had a main class, *Qualifier* (mapped to SUMO *Attribute*), and one subclass for each ICF qualifiers: *Difficulty* (mapped to SUMO *SubjectiveAssessmentAttribute*), *Hindrance* and *Facilitation*. Qualifiers were modelled as enumerated classes, so qualifiers values were declared as individuals of the ICF qualifier classes.

#### Activities and Participation

The ICF component Activities and Participation is meant to express elements used to evaluate a subject functioning against two perspectives, individual and social. The individual perspective is linked to the term activity, the social one to the term participation. Its content consists of a single list of categories for both perspectives, organized in chapters and blocks, and includes areas of life with increasing complexity:

1. Learning and applying knowledge
2. General tasks and demands
3. Communication
4. Mobility
5. Self-care
6. Domestic life
7. Interpersonal interactions and relationships
8. Major life areas
9. Community, social and civic life

Each of the nine areas consists of a series of actions relevant to the area. The actions are usually indicated using the present participle of the corresponding verbs, for example, watching, listening, carrying multiple tasks, communicating, kneeling, washing body parts, preparing meals, etc. More precisely, all the actions listed are performed with a specific purpose, in contrast to unintentional actions like dreaming, trembling or physiological activities that are included in the Body Functions component. In this study the use of the term “intentional” does not denote the explicit will of a person to perform an action but it is used solely to distinguish activities from physiological actions.

The ICF does not distinguish between activities and participation: it gives a single list of actions for both of them without an a priori classification in what is always considered an activity and what is always considered a participation. Furthermore, Activities and Participation component can be used to denote activities, or participation, or both. The fact that in the ICF there is not distinction between activities and participation means that a consensus was not reached on a criterion to objectively classify actions into the two separate concepts. It can be deduced, then, that the classification is subjective, that is it depends on the specific subject under assessment. With the aim of possibly distinguishing participation from activity, in the model we also defined the participation concept as a subclass of activity: in this way, we kept activities and participation together (activity class) but gave the opportunity to specify that an action, for a subject under assessment, has actually social relevance, that is it is a participation.

In order to realize the bridge between ICF and SUMO, the following SUMO class was identified:

- *IntentionalProcess*: a process that has a specific purpose for the agent who performs it.

#### Concepts of the activity context

The subject under evaluation, as the executor of the action, is its agent that purposedly performs the action. Since all the agents in the subject matters are human beings, in the model the class of agents will be called Human. The concept of agent is defined also in SUMO with the class Agent, that has the sub class Human, which was used as the super class of a class with the same name in the ICF model.

To represent the relation between the agent and the action performed, in SUMO there is the generic object property *agent*, whose meaning is to relate a process to its active determinant, either animate or inanimate, who determinates the process with or without voluntary intention. This is a generic relation that expresses exactly what is meant by its name. In SUMO there is a more specific object property between a process and an agent: *hasSkill*. This property expresses the capability of an agent in performing a process with the restriction that this ability practiced can be evaluated to some measurable degree. Since the concept behind *hasSkill* is more specific than that of the agent property, *hasSkill* should be preferred to the generic *agent*. Nevertheless, the object property *agent* has been chosen because of its neutral semantics: an ICF evaluation reveals what a person is able to do but also the actions that a person has difficulty performing, that is he/she has no skill in doing them. For this positive assumption, the implication of ability, the *hasSkill* relation is considered less appropriate than *agent* and has been discarded.

In order to catch the values of Performance and Capacity qualifiers, it should be considered how the subject is executing the action, that is his or her level of difficulty. In other words, the interest is in the way the execution takes place. The SUMO property *manner* expresses a relation between a process and an attribute with the purpose to specify a quality of the process execution. The *manner* object property represents what is usually denoted by adverbs in natural language.

It can be found a clear parallelism between the SUMO object property *manner* and *attribute*. Both of them allow expressing a quality, but *manner* is a property applied to processes while *attribute* is applied to objects. This suggests that the choice to use *manner* and *attribute* object properties is coherent with SUMO definitions and concepts.

The use of the *manner* relation is suitable to associate a difficulty value to an action, but it is necessary to distinguish between performance values and capacity values. To this purpose, two relations between an activity and a difficulty value were defined:

- *performance*: a relation that connects an activity with the difficulty value of the performance qualifier;
- *capacity*: a relation that connects an activity with the difficulty value of the capacity qualifier.

Both of them were defined as sub relations of the *manner* object property of SUMO.

The main concepts identified so far are agent, activity and difficulty. As already seen, the agent should be related to the activity he/she could perform and the performed activity should be related, with two distinct relations, to the difficulty concept, that is the *Difficulty* dataset that, as a concrete domain, holds all the possible values of difficulty.

What is evaluated in terms of difficulty is not “an” action, but “the” action performed by the agent. That is, difficulty applied to this more complex concept, is a quality of the activity when it is performed by a specific agent at the time of the assessment. This is a quaternary relation that holds between the agent, the activity and the two relations toward difficulty values, one for performance and the other for capacity.

To represent the quaternary relation in the ICF model, the design pattern described by W3C [34] has been followed, adapting that pattern to quaternary relations. Of the two possible alternative patterns proposed, it has been chosen to follow the “Introducing a new class for a relation” pattern. Thus, in order to implement the pattern, a new class to represent the quaternary relation concept has been created. The new relation class has been linked to the *Human* class, the *ActivityAndParticipation* class, the *Difficulty* class for the performance qualifier and the *Difficulty* class again for the capacity qualifier. The relation class introduced has been called *QualifiedActivity*. As the *ActivityAndParticipation* class, also the *QualifiedActivity* class represents actions and therefore is a sub class of SUMO *IntentionalProcess*.

**Fig. 4:**
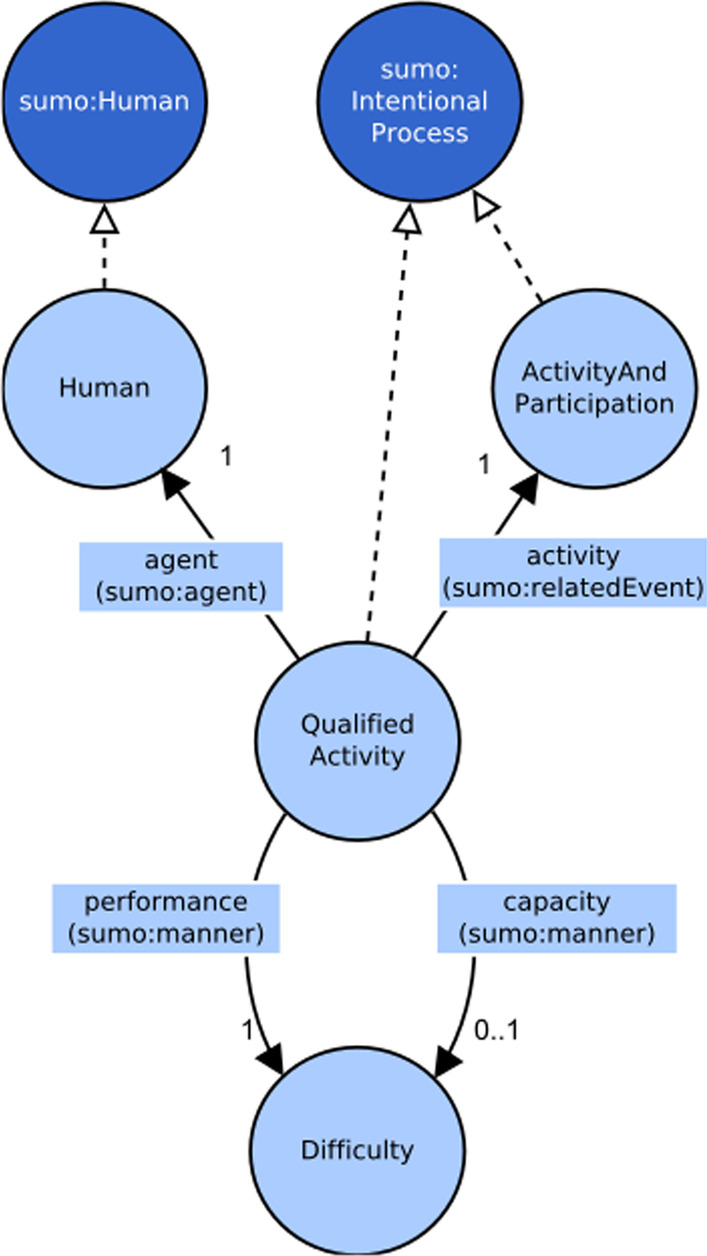
Conceptual model of the activity context

The whole conceptual model of activities is presented in Fig. 4.

#### Participation

The ICF participation definition is “involvement in a life situation”; it represents the societal perspective of functioning. Life situation has no further explanation in [1, 2].

In the ICF, the participative nature of an activity is not predefined and should be derived from some characteristics specified by the model. These characteristics can be taken back to the intention of the subject to participate in a life situation: a participation is an action performed in order to take part to social life. To represent the participation concept, the intention of the subject under assessment should be explicitly expressed.

The search of a SUMO class to be used in the mapping with the “life situation” concept started from the root class, *Entity*, and proceeded down in the SUMO hierarchy. Life situation has been considered a physical entity, not an object one but a process one. So life situation is considered a sub class of the *IntentionalProcess* SUMO class.

To denote the intention of an action the following SUMO property has been used:

- *destination*: specifies the target or goal of a process. The domain is a process, the range is a generic entity, that is everything. It is a generic case role that covers “recipient” and “beneficiary”.

The *destination* property connects the *QualifiedActivity* class with the convenient *LifeSituation* class, in order to represent that the intention of the subject in the execution of the activity is the participation in a life situation. This conceptualization allows to express the subjectivity of participation: what is considered a social activity for a subject, may be regarded as a personal activity for another. This is coherent with the actual position of WHO that has not supplied a predefined classification of actions in personal and social activities.

Given this model, the individuals of the participation class (called *QualifiedParticipativeActivity*) are all the individuals of the *QualifiedActivity* class that have the object property destination toward the class *LifeSituation*: *QualifiedParticipativeActivity* is a defined class while *QualifiedActivity* class is a primitive class.

**Fig. 5:**
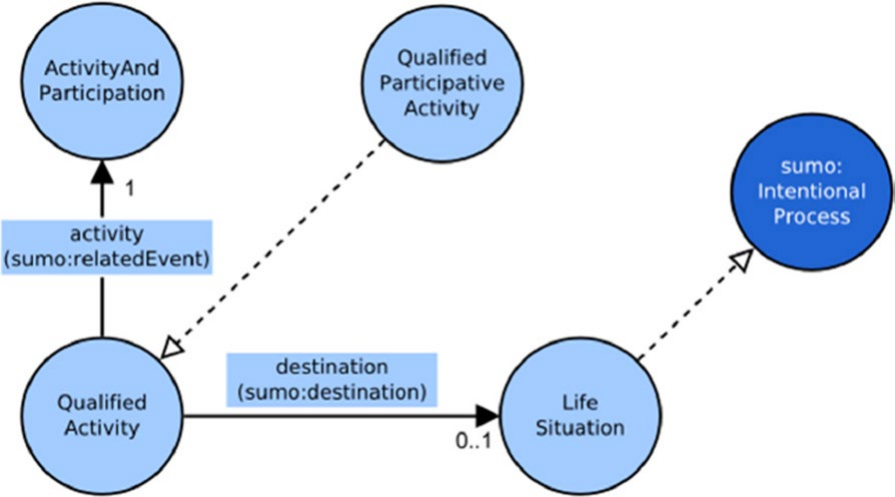
Conceptual model of the participation context

The definition of *QualifiedParticipativeActivity* class in Description Logic would be:
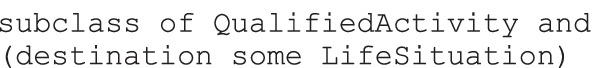


Figure 5 depicts the conceptual model of the *Participation* context. The multiplicity reported in the graph shows that the specification of the destination of a qualified activity is optional: it will be expressed only when the intention of the subject assessed is to participate in a social activity.

#### Contextual factors

The ICF divides the concepts contained in this component into two disjoint sets: environmental factors and personal factors. Both of them represent elements that may have an influence on the ability or health of the subject, but their nature may vary and be very different.

Some observations may be made concerning the mapping of these concepts to SUMO classes. Personal factors represent quality of a human being and can be bound to the SUMO *BiologicalAttribute* class, a sub class of the *InternalAttribute* class. The overlap of the two concepts, personal factor and *BiologicalAttribute*, is not perfect because the ICF meaning of personal factors in some aspects is broader than SUMO *BiologicalAttribute* and in others is narrower. The SUMO definition of *BiologicalAttribute* is “Attributes that apply specifically to instances of *Organism*.” [21]. The reference to a generic organism, that for SUMO is “a living individual, including all Plants and Animals” should be narrowed to only human being. On the other hand, the ICF considers personal factors also aspects like education or profession that are not contemplated by the SUMO semantics. Despite this partial semantic overlap, for the purpose of this study this mapping was judged adequate.

**Fig. 6:**
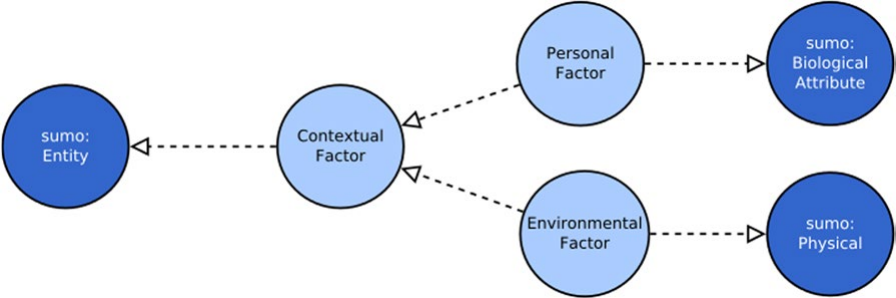
Conceptual model of the contextual factor context

On the contrary, environmental factors are something that physically exist and impact the functioning of a subject as a barrier or a facilitator. So, given this consideration, environmental factors were considered a sub class of the SUMO *Physical* class. The generic contextual factor class is therefore a sub class of the SUMO Entity class. In Fig. 6 the contextual factors high level concepts and their bridge to SUMO classes are depicted. Three classes have been added, *ContextuaFactor*, *EnvironmentalFactor* and *PersonalFactor*. Their mapping to SUMO classes has been defined as well as the hierarchy among them.

#### Defining the influence of contextual factors

Besides the structural characteristics of the contextual factors previously modeled, other important aspects to consider concern their dynamic facets, that is the description of their impact on health states.

There are two aspects to represent: the kind of influence, if positive or negative; the extent or magnitude of the influence. As a first solution, the definition of as many relations as the number of all possible values of the qualifiers could be considered: this is feasible since the number of the values is finite. As a consequence, there would have been six relations for the barrier qualifier and other six relations for the facilitator qualifier:

1. no hindrance
2. mild hindrance
3. moderate hindrance
4. severe hindrance
5. complete hindrance
6. some hindrance
7. no facilitation
8. mild facilitation
9. moderate facilitation
10. substantial facilitation
11. complete facilitation
12. some facilitation

This hypothesis could solve the problem to represent both the aspects of kind and extent of the influence but was not considered appropriate for many reasons: the resulting model would be cluttered with many properties that differ only by the value of the qualifier; the expressiveness of the model would not be clear; qualifiers values would be merged with object properties in a resulting Cartesian product of model elements; the model would be little resilient since any change in qualifiers values would cause a change in the model.

The world to be modeled, that is the influence of the contextual factors, is a relation among factors and health states, where the characteristics of the influence are qualities of the relation between the factor and the influenced element. This is a ternary relation which connects three individuals: an influenced element, a contextual factor and a qualifier value.

As in the previous case, the N-ary relation pattern was implemented introducing a new class for the relation, called Influence. This new class associates:

- the factor that exerts influence, using a new relation with the *ContextualFactor* class;
- alternatively, the extent of the positive influence or the extent of the negative influence, using two relations with the qualifier classes *Facilitation* and *Hindrance* according to the type of influence, positive or negative respectively;
- the entity being influenced with two different relations to specify a facilitating or a hindrance influence.

To implement the last point a new class was introduced, *InfluenceableHealthEntity*, that represents the individuals for which, according the ICF coding rules, can be specified contextual factors influence.

The *Influence* relation class is related to the *InfluenceableHealthEntity* class with two relations: *facilitatedBy*, to express a positive influence, and *hindrancedBy*, to express a negative influence. Both of them are sub relations of *influencedBy*, the relation that was mapped to SUMO.

In addition to the extent or strength of the influence, it is possible to model another feature regarding the contextual factors influence: the origin of the influence. For barrier contextual factors, hindrance could be caused by the presence of the contextual factor or by its absence; for facilitator factors could be considered the accessibility of a resource, and whether access is dependable or variable, of good or poor quality, and so on.

The concept of influence (*Influence* class) accomplishes two roles:

1. it represents the concept of a factor that has the role of influencer of something;
2. it acts as an “interface” between Activities and Participation domain and Environmental Factors domain.

While the second point is particularly relevant because having clear interfaces between parts of a system ensures independence and better resilience, the first allows to connect to the factor all the other concepts that qualify it. Furthermore, it differentiates concept and role of contextual factors, as an important and essential practice in ontology development [35]. In order to express all the characteristics listed before, the Influence class is related to the following classes:

- *InfluenceableHealthEntity*: the ICF element that is influenced by the influencing factor;
- *ContextualFactor*: the root class that represents all contextual factors that may have an influence; thanks to the reference to the *ContextualFactor* class, the model can represent either environmental factors or personal factors;
- *InfluencingManner*: an enumerated class that lists all the possible reasons how the factor is influencing the subject: presence, absence, accessibility, etc.;
- *Hindrance*: the enumerated class of all the values used to measure the extent of negative influence;
- *Facilitation*: the enumerated class of all the values used to measure the extent of positive influence.

The relations defined to associate these elements to *Influence* class are:

- *facilitatedBy*: defines a positive influence between the influenced entity and the factor;
- *hindrancedBy*: defines a negative influence between the influenced entity and the factor;
- *factor*: associates the contextual factor that determines the influence;
- *influencingManner*: specifies the manner of the influence;
- *barrierLevel*: specifies the extent of the negative influence, the value of the hindrance class that qualifies the magnitude of the negative influence;
- *facilitationLevel*: specifies the extent of the positive influence, the value of the facilitation qualifier.

Given this specification, in the model two more classes have been defined, *Facilitator* and *Barrier*, as defined sub classes of *Influence* class using description logic:

- individuals of *Facilitator* class are individuals of *Influence* class which are related to an individual of *InfluenceableHealthEntity* with the *facilitatedBy* relation;
- individuals of *Barrier* class are individuals of *Influence* class which are related to an individual of *InfluenceableHealthEntity* with the *hindrancedBy* relation.

**Fig. 7:**
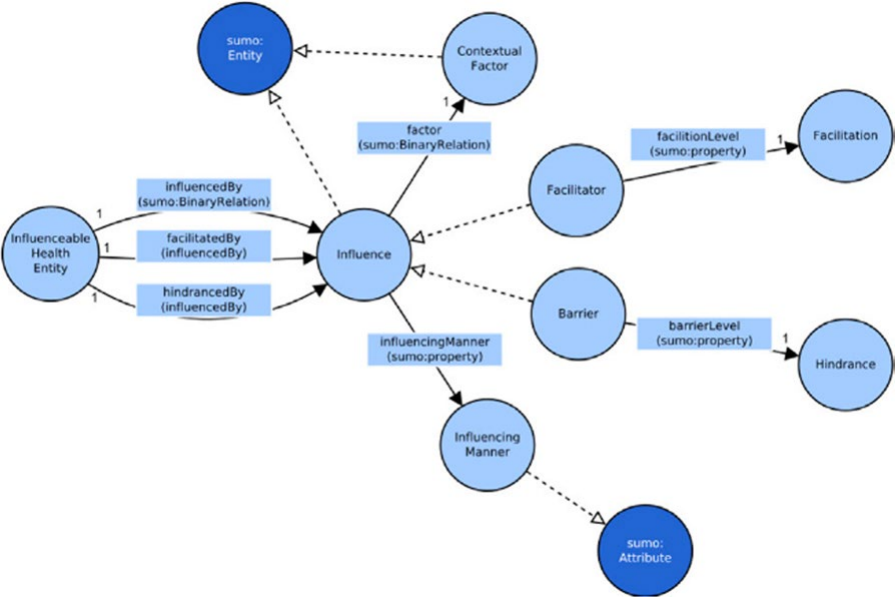
The dynamic aspects of contextual factors

The concepts and their relations concerning dynamical aspects of contextual factors are reported in Fig. 7.

#### Entities influenced by contextual factors

A key element in the conceptual model of the ICF is the modeling of the relations between contextual factors (or environmental factors) and the ICF categories involved in health states. These relations originate from the ICF coding conventions for the Environmental Factors component.

The ICF defines three coding conventions for the environmental factor component:

1. Environmental factors are coded alone, without relating these codes to body functions, body structures or activities and participation.
2. Environmental factors are coded for every component.
3. Environmental factors are coded for capacity and performance qualifiers in the Activities and Participation component for every item.

**Fig. 8:**
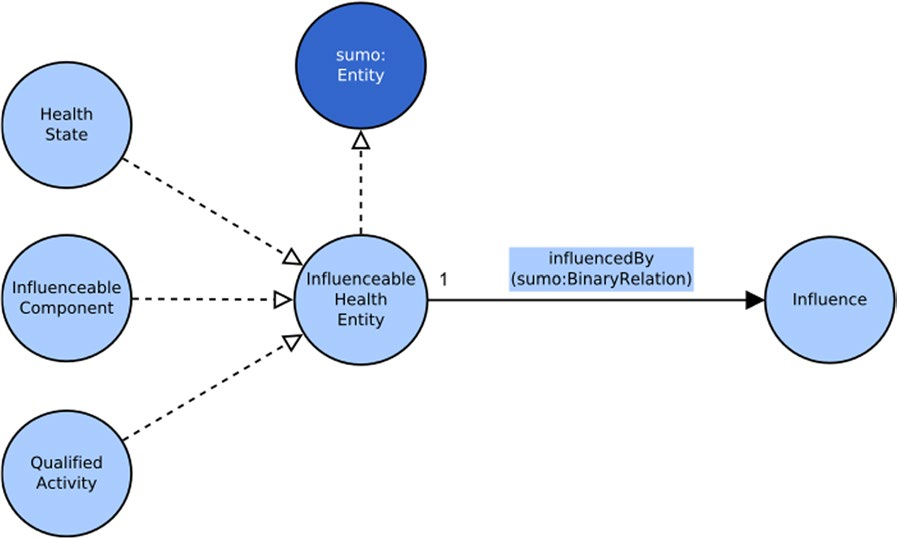
The different associations with contextual factors to model the three coding conventions of ICF

Detailing the work done for modeling the influence in a way that respects these coding conventions is out of scope for this paper, thus we only summarize the results in Fig. 8. The three coding conventions are implemented through the association *influencedBy* with *HealthState* class for convention 1, *InfluenceableComponent* for convention 2 and with *QualifiedActivity* for convention 3.

#### Putting the model together

Using the main concepts that guided the definition of the context map and the partial models defined in the previous paragraphs the conceptual model of the ICF ontology was assembled.

The pillars of the model are three main classes, *ICFCategory*, *Qualifier* and *HealthState*: the first represents the structure of the ICF classification, the second the ICF qualifiers while the third the assessments made using the ICF framework. The relation between these concepts, that is “ICF categories describe health states measured with qualifiers”, was not defined since it is at a very high level and judged general and not very representative.

*ICFCategory* and *Qualifier* classes implement the structure of the ICF, the ICF framework, which was represented in the model with the new class ICF. The relations between the ICF class and the *ICFCategory* and *Qualifier* classes is about composition (it is a whole/part relationship): The ICF class is composed by the *ICFCategory* and *Qualifier* classes and these are part of the *ICF* class.

**Fig. 9:**
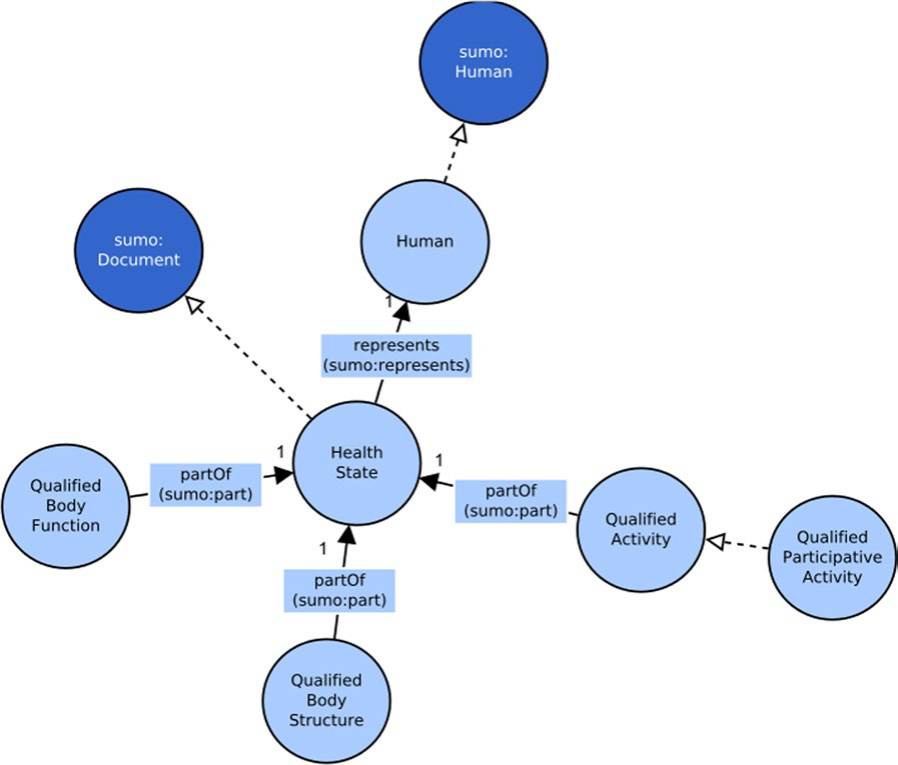
The final conceptual model of the health states bounded context

The third main class, the *HealthState* class, is not part of the ICF class. It consists of the qualified activities (*QualifiedActivity* class) and also of the qualified body functions and qualified body structures not covered in this study. In other words, the *HealthState* class comprises all the assessments made against the health domains categories. The property used to relate these classes to *HealthState* is the *partOf* object property, mapped to the SUMO *part* object property, since all assessments are part of the whole *HealthState*. The multiplicity of these relations is always one because every qualified category describes part of a single health state of a human being (Fig. 9).

**Fig. 10:**
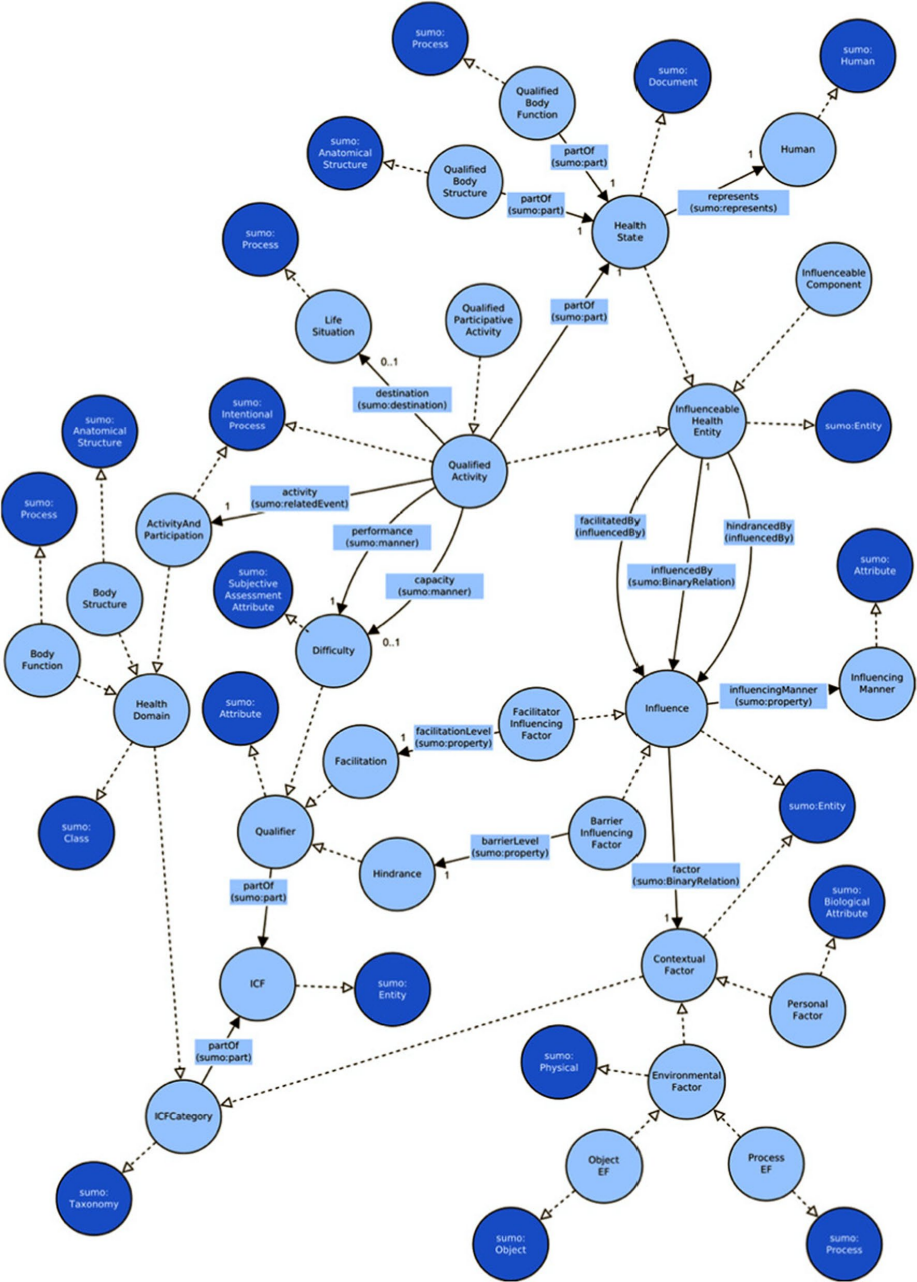
The complete ICF conceptual model

The new association between *QualifiedActivity* and *HealthState* and the relation between *HealthState* and the *Human* class implement an indirect association of the activities to their agent (that is the subject assessed). Therefore, the agent relation previously used to directly link *QualifiedActivity* class to *Human* class, reported in Fig. 3, was dropped. The entire conceptual model, without individuals, is drawn in Fig. 10.

4.5. Conceptual model evaluation.

To evaluate the conceptual model, the ontology validation technique was followed; the goal was to verify that ICF case studies could be represented with the conceptual model before its formalization. The success condition to be satisfied was to express all the semantics of the assertion in model triples.

#### ICF case studies

As a starting point were considered the official training materials and guides that are available to facilitate the use of the ICF in clinical practice. Many of them have been examined from [36–39] where test cases consisted of descriptions of real life situations in terms of a list of observations, where to learning purposes each observation was translated in one or more ICF codes.

In order to test the model, for each evaluation of the test cases considered was identified a specific assertion, or derived one if not provided in the assessment. The assertion was then translated into triples according to the conceptual model. To that purpose, the classes involved in the assertion were initially identified, as well as the relationships that expressed the semantics of the case study. Then appropriate individuals were declared and finally referenced in the model triples.

A total of 60 case studies of this kind were successfully translated into triples; no one gave any issues in the formalization.

As a first example, the following was a case study found in [36]:

E. reacts with some physical aggression (i.e. hitting and spitting) to something that peers do and dislikes or disagrees with (d7202).

The observation could be represented in the ICF model relating an individual of the d7202 sub class of the *ActivityAndParticipation* class to the individual of the *HealthState* class, indirectly, through the *QualifiedActivity* class. In the *HealthState* class there would be an individual that represents the assessment of the person “E.”, which is an individual of *Human*. The individuals involved are listed in Table 1.

**Table 1:** Individuals used to represent the first ICF case study

Class	Individual	Individual short name
D7202RegulatingBehaviours	“Reacting with some physical aggression to something that peers do and dislikes or disagrees with”	Reacting
Human	“E.”	E
HealthState	“E.’s health state 2020_june_6”	E_state_1
QualifiedActivity	“Reacting with some physical aggression in E.’s health state 2020_june_6”	E_state_1_reacting_1

After this analysis, the further step was the translation of the case study assertion into model triples, using the previous individuals and the relations of the conceptual model:
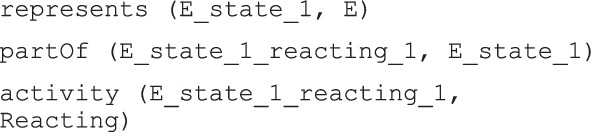


As a second example, one of the many ICF Categorical Profiles reported the coded assessment of Peter, a 20-year-old boy diagnosed with tetraplegia [40]. The profile was a table containing a list of structured evaluations, that is the ICF code and qualifier pairs, where the latter denoted the magnitude of the level of health related to the aspect specified by the ICF code. Part of the ICF profile of the case study is reported in Table 2.

**Table 2:** Excerpt of the ICF profile of the second example

ICF categories		ICF qualifier (problem)
Code	Definition	
…	…	…
d410	Changing basic body positions	1 mild
d4200	Transferring oneself while sitting	2 moderate
d440	Fine hand use	4 complete
d445	Hand and arm use	3 severe
…	…	…

The model triples used to represent the case study are the following:
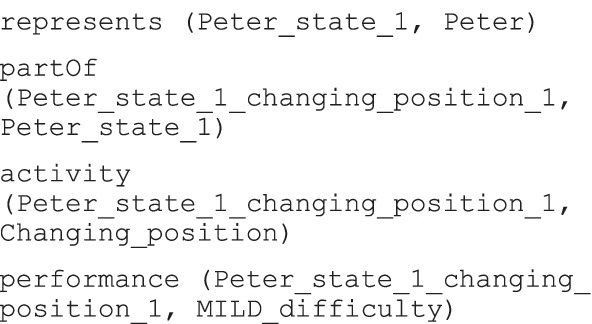


Peter is an individual of *Human* class. The specific state of Peter that was assessed is *Peter_state_1*, an individual of *HealthState* class. *Peter_state_1_changing_position_1* is an individual of the *QualifiedActivity* class, which is related to *HealthState* class with the *partOf* relation. The previous qualified activity is enriched with two pieces of information: the specific activity being evaluated, which is an instance of the *ActivityAndParticipation* class (*Changing_position*), via the *activity* relation; a qualifier of type *difficulty*, that is the instance *MILD_difficulty* of the *Difficulty* class, via the *performance* relation.

**Fig. 11:**
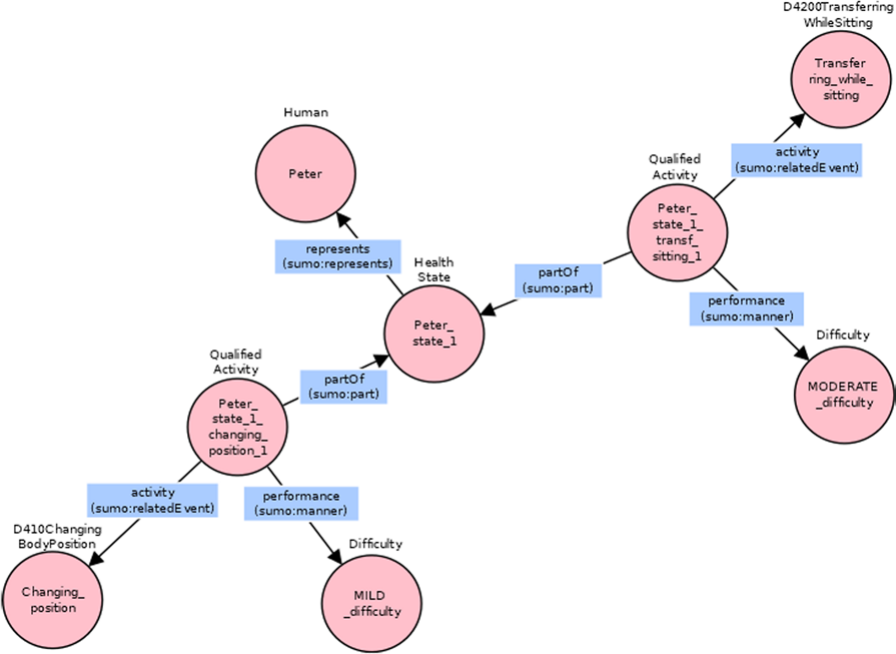
Graphical representation of the first two observations of Peter’s ICF categorical profile

The second observation of the ICF profile was translated in a similar way, with the same relations and classes but using different individuals. Since it refers to the same state of Peter, the first triple, the one used to declare the specific state of Peter under evaluation, coincides with that of the first observation, and it is so for all the observations of Peter’s profile. Figure 11 gives the graphical representation of the first two observations of the ICF profile in Table 2, with corresponding individuals described in Table 3.

**Table 3:** Individuals used to represent the first observation in the ICF profile

Class	Individual	Individual short name
Difficulty	“1 MILD difficulty”	MILD_difficulty
D410Changing BodyPosition	“Changing basic body position”	Changing_position
Human	“Peter Taylor”	Peter
HealthState	“Peter’s health state 2021_mar_17”	Peter_state_1
QualifiedActivity	“Changing basic body position in Peter’s health state 2021_mar_17”	Peter_state_1_changing_position_1

A third example regards an ICF categorical profile from [37], similar to the previous one but with the addition of environmental factors coded using the first convention, that is environmental factors were coded alone, without relating those codes to other codes of body functions, body structures or activities and participation [41]. This was the story of Conrad, a 57-year old Swiss border guard, that sustained a spinal cord injury (SCI) as a result of a surgical intervention. Part of his ICF categorical profile is represented in Table 4.

**Table 4:** Excerpt of the ICF profile of the third example

ICF categories		ICF qualifier (problem)
Code	Definition	
…	…	…
d4153	Maintaining a sitting positions	0 no problem
d4154	Maintaining a standing positions	2 moderate
d465	Moving around using equipment	3 severe
d850	Remunerative employment	4 complete
…	…	…
		Facilitator/Barrier
…	…	…
e1151	Assistive products for personal mobility	3 substantial facilitator
e155	Design, construction of building for private use	3 severe barrier
e580	Health services, systems and policies	2 moderate facilitator
…	…	…

**Fig. 12:**
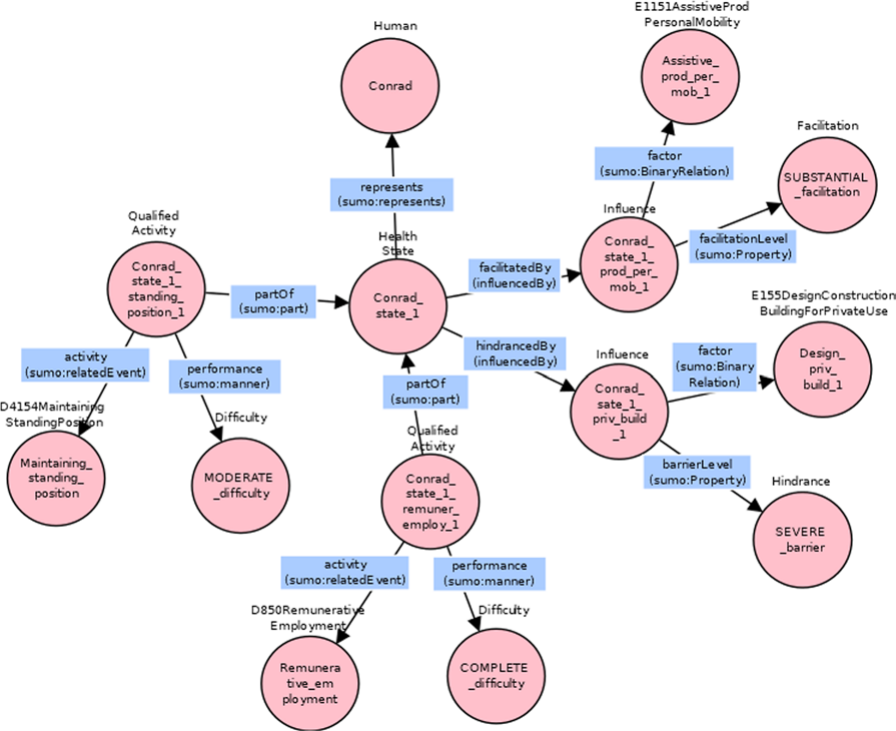
Graphical representation of Conrad’s health state with two activities and two environmental factors

Conrad’s health state is composed by many qualified activities, with their difficult values, and by a set of environmental factors that influence it positively or negatively. In the graph of Fig. 12 is modeled part of this ICF case study: on the left there are two activities and on the right two influencing factors, both of them related to the health state.

The conceptual model was also tested against all the case studies proposed in [39], which provided functional profiles with ICF categories and problem qualifiers for body structures, body functions, activities and participation, complemented with the influence of environmental factors on functioning, indicated by ICF codes and their magnitude as a facilitator or barrier.

#### Ad hoc case studies

Those training scenarios were effective in introducing different healthcare situations for educational purposes but, from a logical point of view, were all simple and similar: they varied only in the values used but the assertions were all of the type category-qualifier. To validate the model more extensively, some complex scenarios were built with the goal to test all the aspects of the model and gain a better coverage of the evaluation phase.

To test the main parts of the model, 3 scenarios were identified:

A. Activities without environmental factors
B. Participation without environmental factors
C. Activities with environmental factors

For each scenario two ad hoc case studies were designed in order to evaluate specific aspects related to it. For all the case studies identified was followed the same validation process seen before and used for the official ICF case studies.

**Fig. 13:**
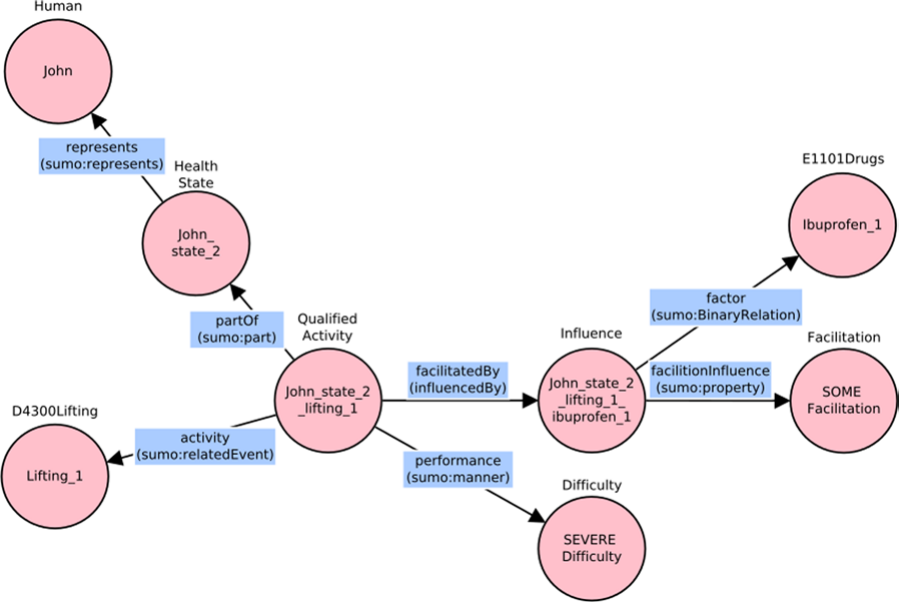
Graphical representation of the sentence in the ad hoc case study

As an example, the scenario C. Activities with environmental factors was meant to investigate if the model could represent the performance of activities in presence of environmental factors. One case study of the scenario was described by the following observation: *“John lifts light small objects with severe difficulty but he has less difficulty if he assumes ibuprofen”*. The qualified action, lifting, is performed with less difficulty with the help of the drug: drugs assumption in the ICF can be considered an environmental factor (e1101), in this case a facilitation. The sentence is vague and does not specify the entity of the influence so the generic value “some” should be coded. Table 5 shows the individuals used to represent the sentence while its graphical representation is depicted in Fig. 13.

**Table 5:** Individuals used to represent the scenario C observation

Class	Individual	Individual short name
D4300Lifting	“Lifting light small objects”	Lifting_1
Difficulty	“3 SEVERE difficulty”	SEVERE_difficulty
Facilitation	“SOME facilitation”	SOME_facilitation
HealthState	“John’s state 2018_feb_4”	John_state_2
Human	“John Smith”	John
QualifiedActivity	“Lifting light small objects in John’s state 2018_feb_4”	John_state_2_lifting_1
E1101Drugs	“Ibuprofen”	Ibuprofen_1
Influence	“Lifting light small objects in John’s state 2018_feb_4 Ibuprofen”	John_state_2_lifting_1_ibuprofen_1

The individuals were used in the following triples:
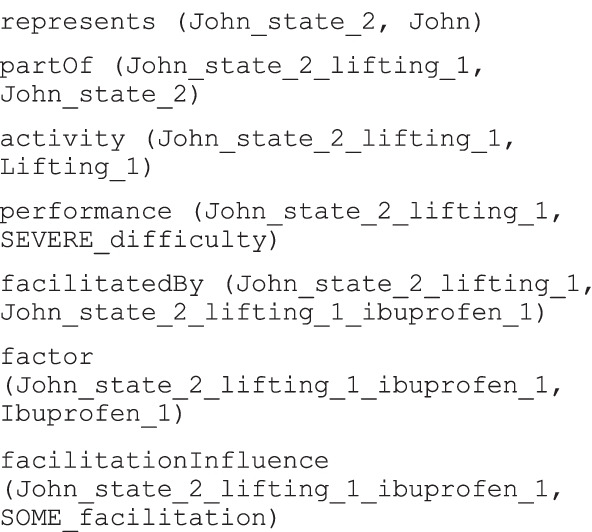


While ICF case studies allowed to evaluate the first coding convention of contextual factors, ad hoc case studies based on sentences have demonstrated the use of more specific parts of the ontology model by verifying the encoding of the influence of environmental factors according to the third coding convention. Sometimes these designed case studies suggested some improvements of the model and were tested until the model was judged adequate to be formalized.

### Formalization

The conceptual model was translated in OWL and afterwards, using its rich semantics, many restrictions were added and the ontology was enriched with description logic constraints.

The implemented ontology contains 63 classes, 48 relations and 81 individuals; 16 classes and 11 relations were involved in the mapping with the SUMO ontology. It contains the whole ICF content model, classes and individuals, but the hundreds of ICF categories for this study were not implemented in corresponding classes and individuals.

#### Graphical model translation in OWL

As a first step toward the formalization of the ICF ontology in OWL, the contents of the conceptual model were translated from the graphical language to OWL. Table 6 lists the conceptual model entities expressed in the VOWL graphical formalism and the corresponding OWL constructs used for their formalization.

**Table 6:** Mapping of conceptual model entities to OWL constructs

VWOL entity	OWL construct
Class	owl:Class
Object property	owl:ObjectProperty
Multiplicity 0..1	owl:FunctionalObjectProperty
Multiplicity 1	owl:ObjectExactCardinality cardinality = "1"

Classes and object properties were created with the descriptive definition documented in the model repository, inserted in the OWL ontology as *rdf:comment*. Object properties were declared with the specification of their domain and range. All the object properties with maximum multiplicity 1 were declared as functional object properties. The functional object properties that had 1 also in the minimum multiplicity were declared with the exact cardinality of 1.

#### Class and object property hierarchies

To better organize classes and relations, some intermediate entities have been added in Protégé. In the newly created ICF ontology there were some cases whereby two relations, with different meaning, associated the individuals of the same couple of classes. These relations had some characteristics in common: equal domain and range, and description of the relationships between the same two classes. In these cases, a third relation was defined, with same domain and range of the other two, that had the role of super property: the two specific object properties became its sub relations.

As an example, the *manner* object property was created as the parent property of *performance* and *capacity*, which are in reality two of its specializations. Another example is the *influencedBy* object property: it denotes that an individual of the domain class undergoes influence by an individual of the *range* class. The two object properties *facilitatedBy* and *hindrancedBy*, which express the nature of the influence, whether positive (facilitation) or negative (hindrance), became sub relations of *influencedBy*.

#### Mapping to SUMO

In order to build the bridge of the formalized ICF ontology with SUMO, the SUMO ontology was imported as a preliminary step. Then, for each class and object property the mapping to the corresponding SUMO entity was formalized: a sub class or sub property relation has been inserted whenever necessary, following what was established in the conceptualization phase.

#### Ontology extension

The OWL ontology was further enriched with the addition of other entities and restrictions. For some properties their inverse was declared: the inverse of *facilitatedBy* was called facilitates, the inverse of *hindrancedBy* was called *hinders*, the inverse of *influencedBy* was called *influences*.

**Fig. 14:**
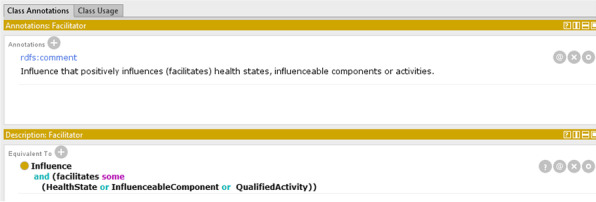
The definition of the Facilitator class in Protégé using the equivalent class constraint

Also the class hierarchy was enriched with the addition of formalized descriptions. As an example, the *Facilitator* class has the following definition: “Influence that positively influences (facilitates) health states, influenceable components or activities”. To be computational, the description should be expressed in a computer understandable language. Using OWL equivalent class constructs, the class is defined as in Fig. 14 where the correspondence between the definition in natural language and in description logic is evident. Analogous definition was provided for the *Barrier* class.

The three classes *HealthState*, *InfluenceableComponent* and *QualifiedActivity* were combined using the boolean operator *or* and the resulting expression is the range of the *facilitates* object property. The *or* boolean operator represents *owl:unionOf* construct, so the range of the *facilitates* object property would be the union of the individuals of the three combined classes.

**Fig. 15:**
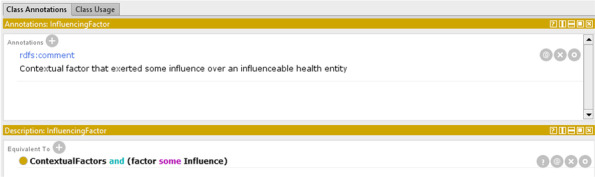
Example of a new class created in the formalization phase

The expressiveness of OWL has been used to add other concepts that were not identified in the conceptualization phase. As an example, the *InfluencingFactor* class has been defined as the class of the contextual factors that exert some influence (Fig. 15).

Other restrictions that were added in the ontology concern the disjointness of the classes, that is the use of the disjoint constraints of OWL. In the ICF ontology disjoint constraints were used in all cases where siblings sub classes implemented a mutually exclusive classification of the individuals of their super class. Some examples are:

- *EnvironmentalFactor* and *PersonalFactor* classes, both sub classes of *ContextualFactor*: a contextual factor is either environmental or personal.
- *ContextualFactor* and *HealthDomain* classes: they are both sub classes of the *ICFCategory* class but a category in the health domain cannot be also a contextual factor and vice versa.
- *ActivityAndParticipation*, *BodyFunction* and *BodyStructure* classes: they are sub classes of the *HealthDomain* class but represent disjoint part of the ICF universe.

## Discussion

In the present paper we explored a possible formalization of the two most distinctive components of the ICF classification: Activities&Participation and Environmental Factors. This formalization might provide the basis for a revision of the classification, in particular when considering the efforts towards the harmonization of the three classifications maintained by WHO: ICD, ICF and ICHI [6]. Joint use of the WHO-FIC classifications is exemplified by the functioning description of an individual whose health condition is classified by ICD and, prospectively, whose planned interventions are coded by ICHI.

The formalization tries to respect the foundations of ICF, which individuates disability as restriction in activities and participation in the interaction between the person’ health state and the environment. It also tries to allow for the different coding styles adopted by the ICF community for representing the Environmental Factors influence.

The formalization stemmed from the official documentation about ICF, which dates back to the finalization of the first version of ICF. However, we are aware that the expert perception of definitions and rules found in the official manual might be changed in time, thus some of the terms we adopted as class names might be considered no more adequate. However, their semantics still represents the basic meaning of the ICF framework, and might provide the basis for an update in line with current understandings of ICF.

One of the themes to emerge from the study is the confirmation of previous research outcomes. Although the overall structure of ICF is well designed, not all formal properties of classifications are respected. The findings suggest that some aspects of the ICF should be addressed and clarified with domain experts and the reference to a shared model of the ICF would be of significant help.

According to the finding of the study, in the ICF area concerning the influence of contextual factors on activities and participations there are interesting possibilities for extending the ICF, as the case of specifying how the influence had been originated. Insights on this topic and future studies can guide the formulation of proposals for the expansion of the semantic wealth of the ICF.

Future work includes the concrete implementation of the ICF categories inside the model, and the extension of the model to the two other components: Body structures and Body functions.

The resulting model, developed in OWL, is available at https://github.com/MITEL-UNIUD/icf-model.

## Conclusions

This study provides a conceptual model for the Activities&Participation and Environmental Factors components of ICF, translated into the logical framework provided by OWL and mapped, when possible, with more general concepts defined in the upper ontology SUMO. The concepts in the model are compatible with the biopsychosocial model of ICF.

This formalization might provide the basis for a revision of the ICF classification in line with current efforts made by WHO on the International Classification of Diseases and on the International Classification of Health Interventions.

## Data Availability

The developed ontological model is available at https://github.com/MITEL-UNIUD/icf-model.
